# Music to improve sleep quality in adults with depression-related insomnia (MUSTAFI): study protocol for a randomized controlled trial

**DOI:** 10.1186/s13063-020-04247-9

**Published:** 2020-04-03

**Authors:** Helle Nystrup Lund, Inge Nygaard Pedersen, Søren Paaske Johnsen, Agnieszka M. Heymann-Szlachcinska, Maryla Tuszewska, Gustav Bizik, Jens Ivar Larsen, Eszter Kulhay, Anelia Larsen, Bettina Grønbech, Helle Østermark, Heidi Borup, Jan Brink Valentin, Jan Mainz

**Affiliations:** 1 Unit for Psychiatric Research, Aalborg University Hospital, Mølleparkvej 10, 9000 Aalborg, Denmark; 2 The Music Therapy Clinic, Aalborg University Hospital, Mølleparkvej 10, 9000 Aalborg, Denmark; 3 Danish Center for Clinical Health Services Research (DACS), Department of Clinical Medicine, Aalborg University and Aalborg University Hospital, Aalborg, Denmark; 4 Unit for Anxiety and Obsession, Aalborg University Hospital, Mølleparkvej 10, 9000 Aalborg, Denmark; 5 Department for Child and Adolescent Psychiatry, Aalborg University Hospital, Mølleparkvej 10, 9000 Aalborg, Denmark; 6 Unit for Depression, Aalborg University Hospital, Mølleparkvej 10, 9000 Aalborg, Denmark; 7 Unit for Bipolar Disorders, Aalborg University Hospital, Mølleparkvej 10, 9000 Aalborg, Denmark; 8 Psychiatry Management, Aalborg University Hospital, Aalborg, Mølleparkvej 10, 9000 Aalborg, Denmark; 9 Department for Community Mental Health, Haifa University, Haifa, Israel

**Keywords:** Music, insomnia, depression, sleep

## Abstract

**Background:**

Insomnia is a common sleep disorder for adults with depression, with major impact on their quality of life. Previous trials suggest that listening to music may be helpful in the treatment of sleep disturbances in healthy populations, including students and elderly. In addition, small studies with clinical populations of traumatized refugees, adults with chronic insomnia and adults with depression insomnia add to the evidence base. However, the impact of music listening in the treatment of depression related insomnia is not well documented.

**Objective:**

To examine the efficacy of music listening on sleep quality, symptoms of depression, and quality of life in adults with depression-related insomnia.

**Method:**

A single-center randomized controlled trial (RCT) in a two-arm parallel-group design is conducted and reported according to the CONSORT guidelines. The trial consists of an experimental group and a standard care control group. Both groups receive standard treatment for depression following Danish clinical guidelines in an outpatient psychiatry unit. The experimental group listens to music for a minimum of 30 minutes at bedtime for 4 weeks.

**Discussion:**

This trial will provide information on the efficacy of music intervention as a non-pharmacological intervention in the treatment of depression-related insomnia. This study will provide novel knowledge concerning music medicine as an evidence-based treatment for depression.

**Trial Registration:**

Clinicaltrials.gov. ID NCT03676491, registered on 19 September 2018.

## Background

Depression is a common health problem and an increasing global burden. WHO has found unipolar depression to be the third largest burden of disease globally in 2004 and is projected to rank first in 2030 [1]. A result of depression is the loss of social and cognitive functions and quality of life. One of the symptoms in depression is reduction in sleep quality (insomnia). Sleep disturbances associated with depression include difficulties in falling asleep and maintaining sleep. Cognitive behavioral therapy (CBT) is recommended in clinical guidelines as first-line treatment [2, 3]. Other treatment modalities for sleep promotion include sleep hygiene, physical activity, light therapy, relaxation techniques, music/nature sounds and acupuncture [4]. Resolving sleep disturbances in patients with active or previous depression is important as it may prevent worsening of symptoms and relapse of depression [1].

Music listening is widely used as a sleep aid [5]. This practice is supported by a recent Cochrane review concluding that music may be helpful in improving sleep quality in insomnia [5, 6]. The review underlines that the small sample sizes of the studies performed so far is a major limitation and concludes that there is a need for additional intervention studies concerning the effect of music listening on insomnia on specific populations, including patients with depression.

Moreover, a systematic review shows that music listening may reduce symptoms of depression in adults when the music listening is conducted regularly for more than 3 weeks [7]. It was reported that patients responded more when given a choice of music [5].

Giving a choice of music and offering a selection of music with a variety to meet individual preferences are both important factors highlighted in research [8, 9]. New research should offer a broad selection of calm music to meet these criteria [6].

Patient-selected music has a risk of being too stimulating (effect-evoking or too dynamic, i.e. increasing pulse and respiratory rate) [10]. Therefore, playlists designed purposefully by music therapists are more likely to have a sleep-inducing effect [8].

Music preference and culture have an influence on the music-listening experience. The perceived quality of the music depends on age, gender, preference, musical training, and cultural belonging [11]. Music listening is a complex experience that influences the individual in many ways, not strictly in relation to sleep and relaxation, but also in relation to wellbeing and sense of self [11]. An improved sense of wellbeing may positively affect the subjective feeling of restedness. The association between wellbeing and quality of sleep is known and reported in the literature [12].

This study aims to add knowledge into these domains, investigating wellbeing by quality-of-life questionnaires as well as subjective and objective measures of sleep quality.

### Feasibility study

We have carried out an observational feasibility study introducing music listening as a non-pharmacological intervention to improve sleep quality for patients with depression [13]. Outpatients diagnosed with unipolar depression and sleeping problems (*n* = 11) were asked to listen to music for at least 30 minutes before going to sleep every night for a period of 4 weeks. Music listening was provided by a sound pillow equipped with an mp3 player containing 10 playlists of 30–60 minutes of relaxing music with different music styles (classical, easy listening, pop, rock) based on the literature of receptive music therapy [14]. The study indicated signs of improved sleep quality [13]. However, the study lacked a control group.

### Rationale for a randomized trial

Effective treatment modalities are in demand to supplement existing treatment of insomnia in depression. The positive effect of music on sleep quality has been reported in a number of study settings with clinical and nonclinical populations [6]. This study contributes to the investigation of the efficacy of music listening in the treatment of depression-related insomnia by combining subjective measures (self-reported data) and objective measures (accelerometer). In addition, a new app with specially designed playlists makes a large selection of music available to meet individual preferences.

### Method – aim and hypothesis

The primary aim is to investigate the effect of music listening on sleep quality at 4 weeks follow-up. The secondary aim is to examine whether music listening is effective in reducing symptoms of depression and improving quality of life as well as improving sleep quality at 8 weeks follow-up in patients with depression-related insomnia.

#### The following hypothesis is tested

**Fig. 1:**
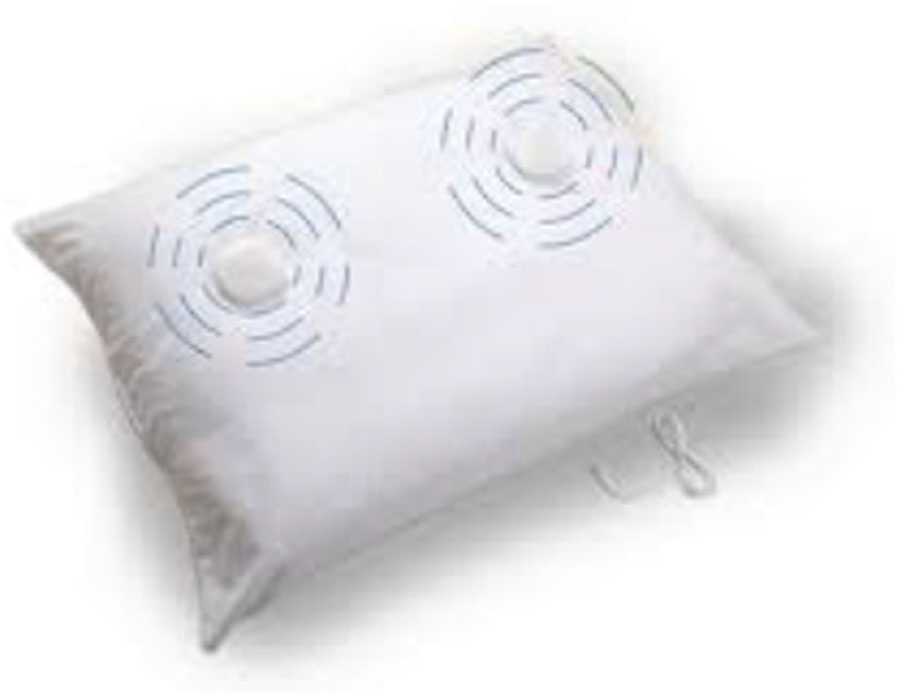
Selected solution-enabling music listening. A sound pillow is a pillow with small internal speakers. The sound pillow features a port through which to connect a player (Mp3/iPad/mobile phone). The user selects the music on the player. (Photo courtesy of Sound Oasis)

**Fig. 2:**
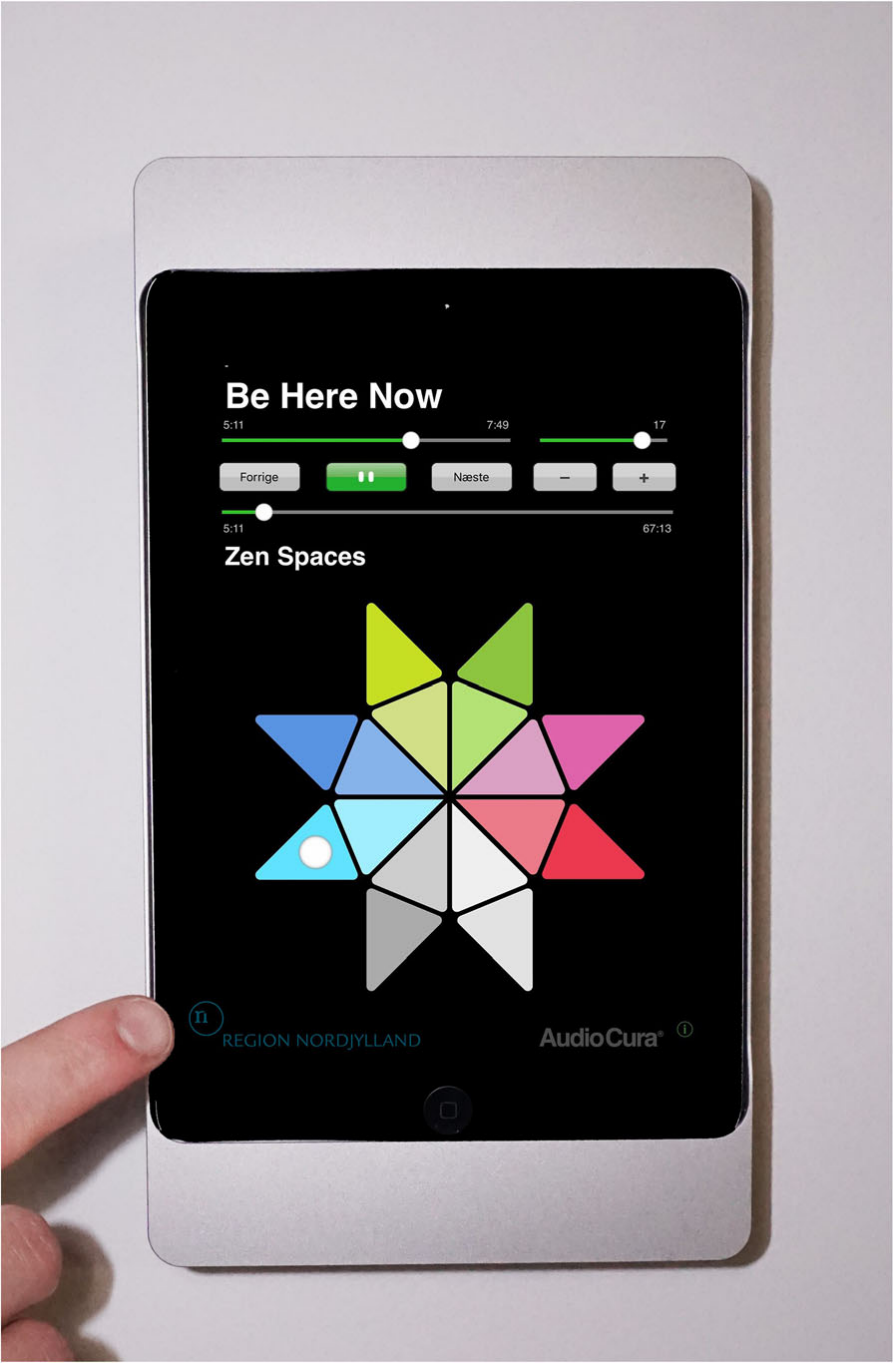
The Music Star is an app developed for iPad to select music from a number of specially designed playlists. Each playlist is represented by a colored triangle forming a star. (Photo courtesy of Musikstjernen IVS)

The controlled use of a sound pillow (Fig. 1) in combination with the Music Star app (Fig. 2) can serve as a sleep aid in reducing depression-related sleep disturbances.

#### Definitions

Music listening, music intervention, and music medicine refer to the use of music as an intervention without an active music therapist. In this protocol, music listening implies listening to pre-recorded music.

Insomnia, sleep disturbances and sleeping problems refer to a broad understanding of the disorder. Symptoms include difficulties initiating and maintaining sleep.

The study is named MUsic STAr For Insomnia (MUSTAFI).

### Trial design

A single-center randomized controlled trial in a two-arm parallel-group design is conducted from May 2018 to December 2019 following the revised Consolidated Standards of Reporting Trials (CONSORT) guidelines [15]. The RCT includes two groups of participants, an experimental group and a waitlist control group. Both groups receive standard treatment for depression following national guidelines.

**Fig. 3:**
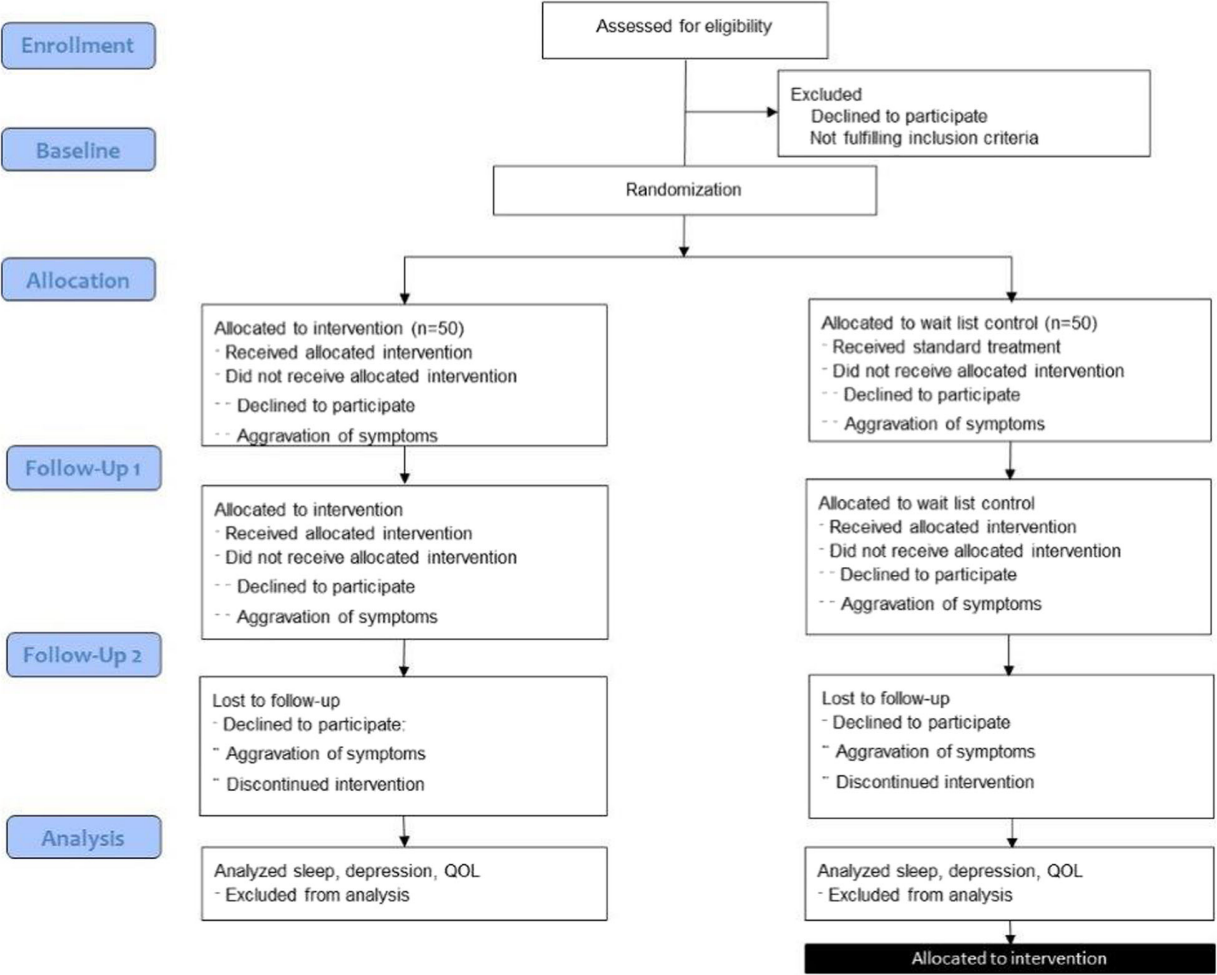
Flow diagram of MUSTAFI phases, including enrolment, allocation, follow-up, and data analysis

The duration of the intervention is 4 weeks. The participants are followed for 8 weeks. Participation takes place in the home of the patient. In addition, three scheduled visits to the hospital in relation to the research project are required. Baseline assessment is performed on the starting day. Follow-up measurements are performed after 4 and 8 weeks (Fig. 3).

**Fig. 4:**
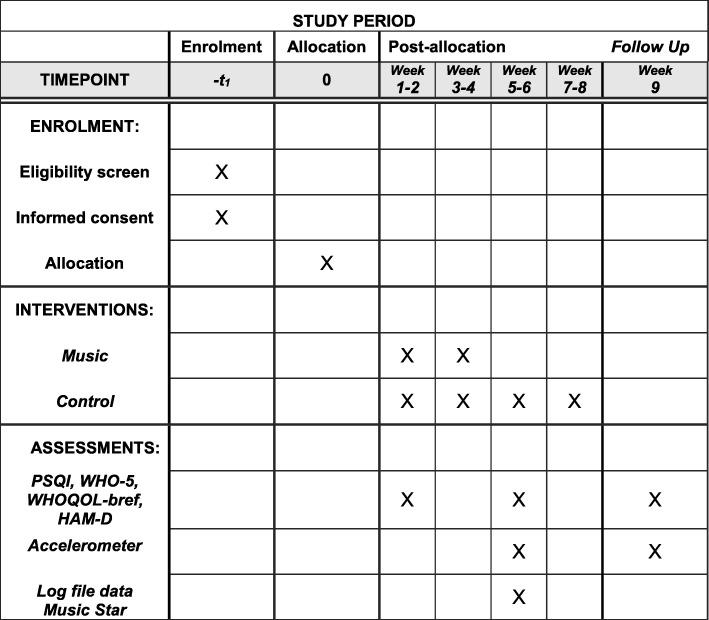
Schedule of enrolment, interventions, and assessments (Standard Protocol Items: Recommendations for Interventional Trials (SPIRIT) flow chart)

The black element in the bottom left of Fig. 3 illustrates the intervention given as an option to the waitlist control group. The participants may take home the sound equipment for 4 weeks. There is no data collection involved. This has been added to the study for ethical reasons and to limit drop out from the control group [16]. The schedule for the trial is described in the SPIRIT flow chart (Fig. 4) and the dimensions of the study protocol have been described, adhering to the SPIRIT checklist (Additional File 1).

### Sample size

Based on a previous study, we assume that the mean decrease in Pittsburgh Sleep Quality Index (PSQI) scale is 3.04 (SD 2) points for the music intervention group and 2.04 (SD 1.67) points for the waitlist control group after 4 weeks of follow-up [17]. When estimating the sample size, we assume that 25% of the eligible patients will be willing to participate in the project. Based on the patient flow in the outpatient clinic and the available resources from the staff recruiting the patients, we assume that a total of 120 participants can be recruited over a period of 18 months. A dropout rate of 20% is expected. Hence, a total of 100 randomized patients are expected to complete the study. Based on the Satterwaite’s two-sample *t* test and with a total of 100 patients (*n* = 50 per group) the power of our study is estimated to be 76% (at a confidence level of 5%).

### Setting and participants

The study will be conducted at a single study site: the Unit for Depression in Psychiatry at Aalborg University Hospital, Denmark. The participants are adult outpatients aged 18–65 years in treatment for unipolar depression in the Northern part of Denmark.

Patients are referred to the unit for depression with different backgrounds. Some patients have not previously been associated with psychiatry and are referred directly from private practice with moderate or severe symptoms of depression. Another group of patients are referred from an inpatient unit. These patients are in recovery and have a stabilized condition in need of follow-up treatment after discharge. A third group of patients have been diagnosed in previous contact or hospitalization and in need for treatment after a recurrent depression.

### Inclusion criteria

All patients are required to have a diagnosis of unipolar depression (ICD-10 Depressive singular episode F32 or Periodic depression F33) and sleeping problems identified by the Hamilton Depression Rating Scale (HAM-D) by a score of at least 2 of a single item or at least 3 in total for the three items (sleep items 4–6). In addition, the patients should receive treatment according to national guidelines for depression. This may include pharmacological treatment, psychotherapy, psycho education and electroconvulsive therapy. Participants are eligible if within age 18–65, in stabilized pharmacological treatment, and having at least 4 weeks of treatment.

### Exclusion criteria

Exclusion criteria are unipolar depression with psychotic episodes, substance or alcohol abuse, or sentence to treatment by law. Patients will be excluded if they have restless legs syndrome, obstructive sleep apnea, or other organic sleep disorders as well as hearing loss. In addition, patients with a dislike of music will be excluded.

### Recruitment

Patients are recruited in the Unit for Depression. Nurses, psychiatrists, and psychologists use the screening tool 4 weeks after the beginning of treatment or after medical treatment is stabilized. Participants are recruited through posters, information by doctors and word of mouth in the unit. After an initial assessment for eligibility, the patient is informed of the option to participate. The recruitment takes place during group meetings or during individual consultation in the unit. When the patient has declared an interest to participate, the psychiatrist of the patient fill out an inclusion document based on information from the patient journal and his/her knowledge of the patient. The document is forwarded to the research leader. If the patient is eligible, the music therapist makes an appointment to give detailed information on participation in the research project.

Patients are included according to the inclusion and exclusion criteria. The patient may be discharged during participation in the research project and finalize as a private person. If the increase in depression symptoms results in hospitalization, the patient may continue participation in agreement with caretakers in the unit. If the patient is changing medication but is interested in participation, the patient will be registered on a waitlist for inclusion.

### Informed consent

The patient may sign the informed consent after receiving oral and written information about the research project. The information explains the aim and the procedures of the research project, the use of sound equipment and accelerometer and the rights as a participant in a scientific health study. The patient has the option to ask questions and if the patient need time to consider the participation, the music therapist offer to call the patient after 1 week and a new meeting is set up for the signing of the informed consent. When the informed consent is obtained and the inclusion criteria are confirmed in the inclusion document, the music therapist proceeds with the randomization. An additional consent for collection and use of log file data from accelerometer and the Music Star app is signed after randomization. Documents of informed consent are kept in a securely locked place.

### Randomization

Randomization takes place when the inclusion document has confirmed eligibility and the informed consent has been signed following the regulations of the The North Denmark Region Committee on Health Research Ethics Randomization is carried out by the use of REDCap (Research Electronic Data Capture) hosted at Aalborg University Hospital. REDCap is a secure web-based application designed to support data capture for research studies. The randomization will be stratified according to age, i.e., under 30 years versus 30 years and above. Randomization is performed using computer-generated block randomization (random block sizes 2–8) with 1:1 allocation between the intervention group and the waitlist control group.

### Procedure

The principal investigator is assigned to give information on the research project to groups and individuals by team members and coming to the unit by appointment. When offering thorough information to obtain informed consent, the music therapist takes the participant to the music therapy clinic situated in a hospital building close by.

### Experimental intervention

The music intervention consists of listening to music with the use of a sound pillow, applying the music from the Music Star app with special designed playlists. The duration of music listening for 30 minutes minimum at bedtime is guided by previous studies [6]. The principal investigator HNL gives an oral guideline to music listening and use of sound equipment at the baseline appointment including how to adjust the volume. The pillow speakers allow audio listening at very low volume. Adherence to the intervention (30 minutes of bedtime music) is monitored through log file data from The Music Star app.

Instructions for the experimental intervention:

- It is required that you listen to music of your choice from the Music Star for at least 30 minutes at bedtime every night for 4 weeks. You may use the Music Star during the night or in the early hours of the morning if you wake up and have difficulty falling asleep again.
- You may turn off the Music Star if you wake up when the music plays and it is disturbing.
- The music will automatically stop when the playlist ends after 30 or 60 minutes.
- You may use the Music Star in the daytime to assist a rest. It is important only to use the Music Star for relaxation and sleep.
- You are advised to listen to the different music available to select your preferred playlists. You may select a particular playlist to play every night. Some people find that it is helpful to let a specific piece of music be a signal for sleep initiation.
- Consider choosing music that you like and that may help you fall asleep.
- You have to listen to music at night in the way it is described for the research purpose, i.e. the sound pillow in combination with the Music Star app.
- You may listen to your own music during the daytime.

Instructions on the use of the sound equipment:

- Place the sound pillow on your bed (remove your own pillow) and place the Music Star close to the bed on a small table or chair for easy access during the night.
- Make sure that you have recharged the Music Star during the day.
- If you have technical problems with the sound equipment, you may contact the research leader by phone from 9am to 12pm on weekdays.

#### Waitlist control group

The participants in the waitlist control group are asked to continue their normal bedtime routines. They are not given any instructions concerning music listening during the 8 weeks. At the baseline appointment, they are informed of the option to take home the music equipment to test for 4 weeks at the end of the 8-week period.

### Instruments

The data are derived from four questionnaires (three are self-reported and one is scored by the psychiatrist, researcher, or project nurse) and log file data from the accelerometer and from the Music Star app. The references concerning validation of each instrument below are selected according to their relevance to this study.

#### The Pittsburgh Sleep Quality Index (PSQI)

Is a commonly used questionnaire measuring self-reported sleep habits in clinical populations in research and clinical practice [18]. A Danish version of the questionnaire will be used. The items refer to sleep habits and disturbances within the last month. The 19 items are divided in seven domains: subjective sleep quality, sleep latency, sleep duration, sleep efficiency, sleep disturbances, daytime dysfunctions and use of antidepressant agents. The seven components form a global score ranging from 0 to 21, each component with a range from 0 to 3. Buysse et al. reported a score of > 5 (indicating poor sleep) yielded a diagnostic sensitivity of 89.6% and a specificity of 86.5% [18]. The scale shows good homogeneity with an internal consistency, with α = 0.83. Acceptable measures of validity were obtained through the ability to distinguish between clinically distinct groups and comparing these with polysomnographic results. A score of > 5 is indicative of severe sleep difficulties in at least two areas, and separating participants in two categories “good” and “poor”. PSQI is validated in psychiatric populations [19].

#### The Hamilton Depression Scale (HAM-D17)

The HAM-D17 consists of 17 items [20]. The items cover the depressive state, the unspecific stress and arousal symptoms, the suicidal thoughts, and lack of insight. The respondent is asked to consider the last 3 days when responding.

Each item in the score is rated from 0 to 4 or 0 to 2, the higher number indication an increase in the symptoms of depression. A guide to the Hamilton rating questioning is used in combination with a rating sheet. The total score indicates the severity of depression symptoms graduating in four categories from unlikely depression to severe depression, with 8–12 indicating minimal depression, 13–17 indicating light depression, 18–24 indicating moderate depression, and 25–52 indicating severe depression.

The 17-item version of the Hamilton Depression Rating Scale is validated [21].

#### The 5-item World Health Organization Well-Being Index (WHO-5)

The 5-item World Health Organization Well-Being Index (WHO-5) is among the most widely used questionnaires assessing subjective psychological well-being [22]. It measures subjective quality of life based on positive mood (good spirits, relaxation), vitality (being active and waking up fresh and rested), and general interest (being interested in things). WHO-5 only contains positively phrased items. The WHO-5 items are: (1) ‘I have felt cheerful and in good spirits’, (2) ‘I have felt calm and relaxed’, (3) ‘I have felt active and vigorous’, (4) ‘I woke up feeling fresh and rested’, and (5) ‘My daily life has been filled with things that interest me’. The respondent is asked to rate how well each of the five statements applies to him or her when considering the last 14 days. Each of the five items is scored from 5 (all of the time) to 0 (none of the time). The score therefore theoretically ranges from zero (absence of well-being) to 25 (maximal well-being). The use of WHO-5 as a measure of severity of depression is validated [24).

#### The World Health Organization Quality of Life BREF (WHOQOL-BREF)

In 1991, the World Health Organization initiated a project with the aim of developing an international, cross-culturally comparable QOL assessment instrument. “It assesses the individual’s perceptions in the context of their culture and value systems, and their personal goals, standards and concerns. The WHOQOL instruments were developed collaboratively in a number of centres worldwide, and have been widely field-tested.” [25]. The WHOQOL-BREF instrument is a shorter version of the original 100-item self-report questionnaire, comprising 26 items measuring four domains: physical health, psychological health, social relationships, and environment. In each question, the respondent reports his/her QOL in the four domains on a 5-point Likert scale. A high score reflects the subjective experience of high QOL. The use of WHO-QOL-BREF is validated [23].

#### The Music Star app

An app designed for iPad in 2014. The Music Star is a self-explanatory user interface to select music from specifically designed playlists. Music therapists have selected the music for the playlists. The app has a built-in log function that registers each event when using the app. The Music Star gives information from log files of music played, time, and duration [24].

The 16 playlists of 30–60 minutes duration are represented by colored triangles forming a star. The four playlists in shades of blue are the most quiet and simple (lowest stimuli), the four playlists in shades of green include some variation and dynamics (moderate stimuli), the four playlists in shades of red have more intensity (highest stimuli). The grey triangles are intended to contain playlists for specific purposes. A grey triangle contains the only non-music playlist of ‘Summer Rain’ consisting of sounds of rain and bird sounds. The rain sound adds a non-music track for the purpose of variety in sound stimuli to meet individual preferences. Two other grey triangles contain special designed playlists for sleep. The classification of music is correlated to the degree of complexity and tension of the musical stimulus based on the taxonomy of music by Wärje & Bonde [25]. The playlists in The Music Star are all categorized in subdivisions of supportive music according to the taxonomy of music. The two sleep playlists have a 30- minute duration. Sleep 1 is a short version of a playlist with music specially composed for relaxation and sleep, including a male voice humming. Sleep 2 is a playlist with a guitar-bass jazz duo playing slow ballads. Sleep 1 and 2 are included for meeting criteria of minimum duration and variety in the music selection. More than 100 music pieces in different genres are available in the playlists.

#### Accelerometer

One triaxial accelerometer with on board memory (Axivity Ax3, Newcastle upon Tyne, UK) placed in a wrist bracelet will be used to measure arm movement during the night. The measurement range was set to ± 8G with a sampling frequency of 25 Hz. A sleep analysis function using a generic algorithm will provide data on sleep duration. Assessing sleep duration with an accelerometer is validated [26].

### Outcome measures

#### Primary outcome measure

We assess subjective quality of sleep through patient self-reports using the PSQI to obtain data on psychological and emotional well-being.

The primary endpoint is defined as change of subjective sleep quality from baseline at 4 weeks. Treatment benefit is achieved when the PSQI score is decreased with statistical significant change after the 4-week intervention period.

#### Secondary outcome measures

We assess sleep quality using actigraphy to obtain objective sleep data. We assess symptoms of depression using HAM-D17 to obtain data on the depressive state. Finally, we assess quality of life using WHO-5 and WHOQOL-BREF to obtain data on psychological well-being considering mood, vitality, and interests (WHO-5) and physical/psychological health, social engagement, and environment (WHOQOL-BREF).

The secondary outcome measures are selected to address these objectives (secondary outcome measure 2, 4, 6, and 8). Measuring change from baseline at 8 weeks, assess sustainability of results, and progression of symptoms over time (secondary outcome measure 1,3,5,7, and 9).

The nine secondary endpoints are defined and listed below:

1. Subjective sleep quality (PSQI): Change of sleep quality from baseline at 8 weeks
2. Objective sleep quality (Actigraphy): Change of sleep quality from baseline at 4 weeks
3. Objective sleep quality (Actigraphy): Change of sleep quality from baseline at 8 weeks
4. Symptoms of depression (HAM-D17): Change of depression level from baseline at 4 weeks
5. Symptoms of depression (HAM-D17): Change of depression level from baseline at 8 weeks
6. Quality of life (WHO-5): Change of quality of life from baseline at 4 weeks
7. Quality of life (WHO-5): Change of quality of life from baseline at 8 weeks
8. Quality of life (WHOQOL-BREF): Change of quality of life from baseline at 4 weeks
9. Quality of life (WHOQOL-BREF): Change of quality of life from baseline at 8 weeks

All outcome measures are collected at baseline, after 4 weeks experimental/wait-list control and again after 4 weeks of follow-up. In addition, registration of pharmacological treatment during the period of participation is included in the data collection.

### Demographics

At baseline, demographic information including age, sex, ethnicity, and partnership, children living at home, handedness, diagnosis code for present depression, first diagnosis of depression, and first psychiatric diagnosis will be gathered. In addition, the participant may add self-reported other disease and/or diagnosis. Work status and education, alcohol use, and habitual use of music for sleep will be registered.

### Statistical methods

We initially perform a descriptive analysis on age, gender, medication, illness duration etc. The two groups are compared within groups and between groups using F-tests and chi-squared tests. Additionally, we compare the groups on outcome measures at baseline.

Data analysis for the RCT will be performed using mixed effects linear regression with subject specific random effects. The intervention group will be initially compared with the control group on change of all outcome measures after 4 and 8 weeks. Age and gender will be added as covariates in all regression analyses. Repeated-measures analysis of variance (RM-ANOVA) will be used to compare results from baseline follow-up one and follow-up two.

Finally, a descriptive analysis of the playlists is included in the study, i.e. analysis of playlist preferences (style, duration, time of day) in the group of patients benefitting most from treatment compared to the group of patients benefitting the least.

Blinding of the researchers performing the scorings before and after the treatment as well as the subjects of the study will not be possible. Patients will be anonymized when analyzing data. All main analyses will be carried out based on the intention-to-treat principle. Information on adherence is obtained from the Music Star log file data. Compliance rate will be part of the primary study publication.

In case treatment is associated with dropout rate, we will investigate differences in baseline characteristics between complete cases and patients that are lost to follow-up. Any baseline characteristics that are significantly different between these two groups will be used as adjustment parameters in a sensitivity analysis of music listening treatment on the primary outcome.

A multiple comparisons analysis will not be performed, since the results of the analyses of music listening treatment on secondary outcome are purely exploratory, in which case multiple testing analysis is less important [26].

### Data collection and management

Data collection consists of several elements. The PSQI, WHO-5, and WHOQOL-BREF are questionnaires filled out exclusively by the participants. The HAM-D rating is performed by the researcher (HNL). Data management is carried out by the use of REDCap. Double data entry is performed by research assistants.

### Ethics

The study is approved by the local ethics committee (N-20170055) and registered by the Danish Data Protection Agency (ID 2017–236). A yearly report of adverse events and other unintended effects of the trial will be obtained by the local ethics committee.

### Dissemination

Results of the study will be published in open-access peer-reviewed scientific journals and presented at international scientific conferences regardless of the conclusion. Broadcast of findings on Danish radio and TV media will be planned through the study.

## Discussion

The aim of this RCT is to investigate the efficacy of music medicine compared with standard care. In order to increase the clinical value of the research, the inclusion and exclusion criteria have been selected to target a group of depressive patients who have serious sleep disturbances. This group of patients is less likely to respond to interventions such as cognitive behavioral therapy. Further, there is an augmented risk of relapse and suicide [27, 28]. Sleep disturbances in depression result in a slowdown of the recovery process for the individual and this is costly for the individual and for the society [12].

If music medicine using the Music Star app combined with a sound pillow shows to be effective in improving sleep, it may not only have a positive effect on sleeplessness but also promote compliance with treatment and increase overall functioning [27].

### Limitations

The primary outcome measure is self-reported sleep. Negative thinking is a symptom in depression that may influence self-rating. The HAM-D17 rating is not performed consistently by one person in all cases. Inter-rater reliability has not been tested and may cause bias.

This study does not consider issues of comorbidity. For some patients, sleep disturbances have been present long before the onset of depression. In this study, sleep disturbances are associated with depression although the comorbid presentation of depression and insomnia may be complex. The analysis of secondary outcomes is exploratory and thus more studies will be needed to confirm the results.

### Trial status

Recruitment will take place from 23 May, 2018 to the end of February 2020. By May 2019, 42 participants had been included in the study. The current protocol is version 3, dated 17 October, 2018.

## Supplementary information

**Additional file 1.**

## Data Availability

The data sets used during the current study are available from the corresponding author on reasonable request.

## Abbreviations

HAM-D: Hamilton Depression Rating Scale
PSQI: Pittsburg Sleep Quality Index
WHO-5: World Health Organization-Five Well-Being Index
WHOQOL-BREF: World Health Organization Quality of Life BREF questionnaire
RCT: Randomized controlled trial
REDCap: Research Electronic Data Capture

## References

[1] The World Health Organization. The global burden of disease: 2004 update Part 4. Glob Burd Dis 2004 Updat. 2008;40–51.

[2] Sundhedsstyrelsen. National Klinisk Retningslinje for Unipolar depression. 2016..

[3] Riemann D, Baglioni C, Bassetti C, Bjorvatn B, Dolenc Groselj L, Ellis JG (2017). European guideline for the diagnosis and treatment of insomnia. J Sleep Res.

[4] Chen J, Liu JH, Xu N, Liang ZH, Xu ZH, Xu SJ (2013). Effects of acupuncture treatment on depression insomnia: a study protocol of a multicenter randomized controlled trial. Trials.

[5] Trahan T, Durrant SJ, Müllensiefen D, Williamson VJ (2018). The music that helps people sleep and the reasons they believe it works: a mixed methods analysis of online survey reports. PLoS One.

[6] Jespersen K, Koenig J, Jennum P, Vuust P (2015). Music for insomnia in adults. Cochrane Database Syst Rev.

[7] Chan MF, Wong ZY, Thayala NV (2010). A systematic review on the effectiveness of music listening in reducing depressive symptoms in adults. JBI Libr Syst Rev.

[8] Short A, Ahern N (2009). Evaluation of a systematic development process: relaxing music for the emergency department. Aust J Music Ther.

[9] Bonde LO (2008). Playlists and patients’ preferences: comments on short & aherns article “evaluation of a systematic development process: relaxing music for the emergency department”. Aust J Music Ther.

[10] Bradt J, Dileo C, Grocke D (2010). Music interventions for mechanically ventilated patients. Cochrane Database Syst Rev.

[11] MacDonald R, Kreutz G, Mitchell L. What is music, health and wellbeing and why is it important. Music Heal Wellbeing. 2012:2–12.

[12] Garbarino S, Lanteri P, Durando P, Magnavita N, Sannita WG (2016). Co-morbidity, mortality, quality of life and the healthcare/welfare/social costs of disordered sleep: a rapid review. Int J Environ Res Public Health.

[13] Lund HN, Pedersen IN. Pilot project: sound pillow treatment to improve sleep quality for patients with depression or bipolar diagnosis with sleeping problems. 24th Eur Congr Psychiatry, EPA 2016 Madrid Spain. Elsevier Masson SAS; 2016;33:S80.

[14] Grocke DE, Wigram T (2007). Receptive methods in music therapy: techniques and clinical applications for music therapy clinicians, educators, and students.

[15] Altmann DG, Schulz KF, Moher D, Egger M, Davidoff F, Elbourne D (2001). The revised CONSORT statement for reporting randomized trials: explanation and elaboration. Ann Intern Med..

[16] Bradt J (2012). Randomized controlled trials in music therapy: Guidelines for design and implementation. J Music Ther.

[17] Deshmukh AD, Sarvaiya AA, Nayak AS (2009). Effect of Indian classical music on quality of sleep in depressed patients: a randomized controlled trial. Nord J Music Ther.

[18] Buysse DJ, Reynolds CF, Monk TH, Berman SR, Kupfer DJ (1989). The Pittsburgh sleep quality index: a new instrument for psychiatric practice and research. Psychiatry Res.

[19] Liu XC, Tang MQHL (1996). Reliability and validity of the Pittsburgh Sleep Quality Index. Chinese J Psychiatry.

[20] Hamilton M (1960). A rating scale for depression. J Neurol Neurosurg Psychiatry.

[21] Bobo WW, Angelero GC, Jenkins G, Hall-Flavin DK, Weinshilboum R, Biernacke JM (2016). Validation of the 17-item Hamilton depression rating scale definition of response for adults with major depressive disorder using equipercentile liniking to clinical global impression scale ratings: analysis of pharmacogenomic research network antidepress. Hum Psychopharmacol.

[22] Topp CW, Østergaard SD, Søndergaard SBP (2015). The WHO-5 wellbeing index: a stystematic review of the literature. Psychother Psychosom.

[23] Lund HN, Bertelsen LR, Bonde LO (2016). Sound and music interventions in psychiatry at Aalborg University. Sound Eff.

[24] Van Hees VT, Sabia S, Anderson KN, Denton SJ, Oliver J, Catt M (2015). A novel, open access method to assess sleep duration using a wrist-worn accelerometer. PLoS One.

[25] Warja M, Bonde LO (2014). Music as co-therapist: towards a taxonomy of music in therapeutic music and imagery work. Music Med.

[26] Li G, Taljaard M, Van Den Heuvel ER, Levine MAH, Cook DJ, Wells GA, et al. An introduction to multiplicity issues in clinical trials: the what, why, when and how. Int J Epidemiol. 46:746–55. 10.1093/ije/dyw320 28025257

[27] Fava M (2004). Daytime sleepiness and insomnia as correlates of depression. J Clin Psychiatry.

[28] Jindal RD, Thase ME (2004). Treatment of insomnia associated with clinical depression. Sleep Med Rev.

